# Clinical Features and Outcomes of 124 Italian Patients With Treatment Resistant Depression: A Real-World, Prospective Study

**DOI:** 10.3389/fpsyt.2021.769693

**Published:** 2021-11-05

**Authors:** Giulio Perugi, Paola Calò, Sergio De Filippis, Gianluca Rosso, Antonio Vita, Marina Adami, Giuseppe Ascione, Joachim Morrens, Dario Delmonte

**Affiliations:** ^1^Department of Clinical and Experimental Medicine, University of Pisa, Pisa, Italy; ^2^Mental Health Department, Azienda Sanitaria Locale Lecce, Lecce, Italy; ^3^Neuropsychiatric Clinic, Villa Von Siebenthal, Genzano di Roma, Italy; ^4^Department of Neurosciences ‘Rita Levi Montalcini’, University of Torino, Turin, Italy; ^5^San Luigi Gonzaga University Hospital of Orbassano, Orbassano, Italy; ^6^Department of Clinical and Experimental Sciences, University of Brescia, Brescia, Italy; ^7^Department of Mental Health and Addiction Services, Spedali Civili Hospital, Brescia, Italy; ^8^Janssen Italy, Cologno Monzese, Italy; ^9^Janssen EMEA, Beerse, Belgium

**Keywords:** treatment resistant depression, treatment outcome, health-related quality of life, cohort study, treatment pattern, real-world study, major depressive disorder

## Abstract

**Introduction:** Treatment-resistant depression (TRD) is a debilitating condition affecting 20–30% of patients with major depressive disorders (MDD). Currently, there is no established standard of care for TRD, and wide variation in the clinical approach for disease management has been documented. Real-world data could help describe TRD clinical features, disease burden, and treatment outcome and identify a potential unmet medical need.

**Methods:** We analyzed the Italian data from a European, prospective, multicentric, observational cohort study of patients fulfilling TRD criteria by the European Medicine Agency, with moderate to severe major depressive episode, and starting a new antidepressant treatment according to routinary clinical practice. They were followed up for minimum 6 months. Treatments received throughout the study period, disease severity, health-related quality of life and functioning were prospectively recorded and analyzed.

**Results:** The Italian subcohort included 124 TRD patients (30.2% of patients of the European cohort; mean age 53.2 [sd = 9.8], women: 82, 66.1%). At enrollement, the mean (SD) duration of MDD was 16 years (sd = 11.1) and the mean duration of the ongoing major depressive episode (MDE) was 97.5 weeks (sd = 143.5); low scores of quality of life and functioning were reported. The most frequently antidepressant classes started at baseline (data available for 98 subjects) were selective serotonin reuptake inhibitors (SSRI, 42 patients [42.9%]) and serotonin-norepinephrine reuptake inhibitors (SNRI, 32 patients [32.7%]). In terms of treatment strategies, 50 patients (51%) started augmentation therapies, 18 (18.4%) combination therapies and 24 (24.5%) monoterapies (6 patients [6%] started a non-antidepressant drug only). Fourteen patients (11.3%) were treated with a psychosocial approach, including psychotherapy. After 6 months of treatment, clinical assessments were collected for 89 patients: 64 (71.9%) showed no response, 9 (10.1%) response without remission and 16 (18.0%) were in remission; non-responder patients showed lower quality of life and higher disability scores than responder patients.

**Conclusions:** In our sample of TRD patients, we documented substantial illness burden, low perceived quality of life and poor outcome, suggesting an unmet treatment need in TRD care in Italy.

**Registration Number:** ClinicalTrials.gov, number: NCT03373253.

## Introduction

Major depressive disorder (MDD) is a highly prevalent and burdensome mental disorder (1). The lifetime risk of MDD ranges 5–18% in the general population, with considerable variations across countries (2, 3). A recent publication from the World Health Organization (WHO) reported a prevalence of about 5% for depression in the European region (4). MDD is associated with social withdrawal, functional and vocational impairment, medical morbidity and increased utilization of health care services (1). According to WHO, MDD is the main contributor to the global years lived with disability worldwide (4).

While the therapeutic goal of MDD is remission with complete functional recovery (5), a sizeable subgroup of patients reports residual or persistent symptoms of depression despite receiving evidence-based antidepressant treatments (6). Evidence indicates that the likelihood of remission decreases with consecutive treatment lines (7). In the large, US, multi-step Sequenced Treatment Alternatives to Relieve Depression (STAR^*^D) study there was a significant reduction of remission chances after the second and the third treatment steps. Remission rates were 36.8% for the first and 30.6% for the second treatments, while in patients who had failed the first two treatments, the remission rate dropped to 13.7% with the third and 13% with the forth treatment steps (7). A similar pattern was observed for response rates. In addition the greatest illness burden (i.e., depression chronicity, psychiatric or general medical comorbidity) was characteristic of those who required more treatment steps.

According to the European regulatory authority, treatment resistant depression (TRD) is defined as a major depressive episode (MDE), in the context of MDD diagnosis, failing to achieve adequate response after treatment with at least two antidepressants given at adequate dose and duration (8). With this definition, TRD affects 20–30% of MDD patients (9). Other staging methods for TRD consider different parameters, precluding consensus on the prevalence of the condition (10–12).

Available evidence indicates that TRD patients experience a dramatically higher burden of illness compared to patients who respond to treatment, with more severe symptomatology, poorer quality of life, higher disability, reduced life expectancy, greater frequency of suicide attempts, and higher health care costs (13–15).

Since there are no approved drugs in Europe for the treatment of TRD, current pharmacological options in TRD are as much the same as those available for the treatment of MDD (1), and include selective serotonin reuptake inhibitors [SSRI], serotonin-norepinephrine reuptake inhibitors [SNRI], tricyclic antidepressants [TCA], monoamine oxidase inhibitors [MAOI], and atypical antidepressants (16, 17). Esketamine, in conjunction with a SSRI or SNRI, is the only drug recently approved specifically for TRD in Europe (2019) (18). Among non-pharmacological treatments, psychotherapy and neurostimulation therapies, such as transcranial magnetic stimulation (TMS) have been proposed, usually in combination with oral medications. However, their efficacy in TRD is still questionable (19). Electroconvulsive therapy (ECT) is highly effective but is not widely available in Italy (20). Other non-pharmacological approaches such as deep brain stimulation (DBS) and vagal nerve stimulation (VNS) are invasive, with safety and tolerability concerns (21).

In clinical practice, common treatment strategies to tackle non-response in MDD involve switching to an alternative antidepressant, adding an antidepressant (combination therapy) or adding an augmentation agent (usually lithium, second-generation antipsychotics [SGA] and thyroxine) to the ongoing pharmacological therapy (22). Combining antidepressants or augmenting with non-antidepressant medications are typically employed when patients achieve partial response to the initial medication regimen; however, combined therapy may be associated with more severe side effects and poor tolerability. In the case of non-response and/or poor tolerability of the initial therapy, switching to another antidepressant is a common clinical choice (23). An alternative approach is to combine common drugs with psychotherapy or neurostimulatory treatments. In any case, the optimal treatment sequence in TRD is yet to be defined.

Real-world evidence on patients with TRD is scarce (15, 24, 25), especially from European countries (26), and mainly derived from retrospective studies based on administrative databases or electronic health records (27–29). Within these studies, data on prescribing patterns and illness outcome are sparsely reported (24–26, 30). Real-world data are of paramount importance since patients included in randomized clinical trials (RCTs) of depression are poorly representative of the treatment-seeking depressive patients in routinary clinical practice. Indeed, patients included in RCT are relatively uncomplicated, without relevant medical and psychiatric comorbidities, with absent suicidality, and higher likelihood of responsiveness, as reflected by the high rate of placebo response (31). In addition, in the clinical trial setting patients are more extensively assessed and more frequently monitored than in clinical practice, and treatment adherence is not a major issue (32). Real-world studies in the MDD/TRD context are therefore needed to fill the gap between clinical research and routinary clinical practice.

In light of this need, a cohort study was established in 2018 to collect real-world data from TRD patients treated in clinical practice in several European countries (33, 34). Available data of 411 TRD patients with moderate-to-severe disease starting a new antidepressant treatment and followed for a minimum 6 months period were analyzed. Results from the overall European cohort showed the substantial impact of TRD on patients and society (33), and underlined the wide range of treatments used in clinical practice and the poor response rates of these patients (34).

Given the lack of a widely shared and consolidated standard of care and the possible difference among countries in the clinical approach to TRD, it is important to describe the country specific approach and prospective outcome in the management of TRD.

Therefore, the present investigation is aimed to describe the socio-demographic and clinical characteristics, treatment patterns and outcomes of the Italian patients included in the large European TRD cohort, considering that about one third of the patients of this cohort were recruited in Italy. The healthcare resource use of these patients was also documented.

## Materials and Methods

### Study Design and Participants

The present study is based on the Italian component of a European, prospective, multicentric, observational cohort study of TRD patients (ClinicalTrials.gov number, NCT03373253). Study methods for the overall European cohort have been previously described (33). The study was conducted in the period February 2018 (first patient in) and January 2020 (last visit of the last patient). Briefly, in- and outpatients aged 18 to 74 years, of both genders, with a diagnosis of MDD (according to the Diagnostic and Statistical Manual of Mental Disorders Fifth Edition [DSM-5] or the International Statistical Classification of Diseases and Related Health Problems, 10th Revision [ICD-10] criteria for MDD or Depressive disorder) and fulfilling TRD criteria by the European Medicines Agency (EMA) (i.e., treatment failure on at least 2 different oral antidepressants, given at adequate dose and duration, as evaluated by the Massachusetts General Hospital-Antidepressant Treatment Response Questionnaire [MGH-ATRQ]) were enrolled in the study. To be enrolled, patients had to have moderate-to-severe depressive episode, as defined by a Montgomery-Åsberg Depression Rating Scale (35) (MADRS) ≥20 and needed to be initiating a new treatment regimen for MDD (i.e., any pharmacological or non-pharmacological treatment, including neurostimulation and psychotherapeutic interventions, prescribed to replace, or in addition to, the previous treatment; switches to biosimilars or changes in dose were not considered as new treatments) according to standard care; treatment choice, dose and method of administration was at the discretion of the prescribing physician.

Patients were excluded if they had: current or past psychotic disorders, MDD with psychotic features, bipolar disorders or intellectual disability according to DSM-5 or ICD-10; history of suicidal behavior within 1 year prior to enrolment; homicidal or suicidal ideation with intent within 1 month prior to entering the study; history of moderate or severe substance disorder (including alcohol) within 6 months prior to enrolment.

TRD patients were followed-up for a 12-month observation period with a minimum of 6-months and an extended observation period up to 6 months after enrollment of the last patients (end of study). In the overall cohort, patients were enrolled in multiple centers from Belgium (9 sites, 44 patients), Germany (23 sites, 59 patients), Italy (22 sites, 124 patients), the Netherlands (3 sites, 27 patients), Portugal (10 sites, 37 patients), Spain (16 sites, 71 patients) and the United Kingdom (15 sites, 49 patients); in the present study, the Italian sub-cohort was analyzed.

The study was conducted according to the Declaration of Helsinki, with the approval of the study Local ethics review boards. All patients provided written informed consent to participate in the study; their capability to give consent was judged by the treating physician.

### Data Collection

Data were collected at baseline, at scheduled visits (every 6 months), and at study end. Data were also collected in case of premature study withdrawal and clinically relevant events, such as a change in treatment, admission to – or discharge from – inpatient care, symptoms relapse or MDE remission. Individual participant information was mainly recorded from patient's medical records; patients were also asked to complete questionnaires for the self-assessment of quality of life and functionality.

At baseline, patient's socio-demographic characteristics, history of the depressive disease, treatment history for the current MDE, and comorbidities were recorded. Clinical evaluation was performed at baseline and at each visit. Depression severity was assessed through the MADRS (35) (range: 0–60, with higher scores for higher depressive symptomology) and the Clinical Global Impression of Severity (CGI-S) (36) (range: 1–7, with higher scores for more severe mental illness). Change in depression severity was evaluated through the Clinical Global Impression of Change (CGI-C) (36) (range: 1–7, with lower scores for improvement and higher scores for worsening of depression). Patient's quality of life and functioning was collected at baseline and scheduled visits. Quality of life was assessed by the self-reported EuroQol 5-dimension 5-level (EQ-5D-5L) instrument (37). The EQ-5D-5L questionnaire assessed five dimensions (i.e., mobility, self-care, usual activities, pain/discomfort and anxiety/depression), with five levels of functioning (e.g., no problems, slight, moderate, severe and extreme problems). Results on each dimension were converted into a single utility index (generally ranging 0–1, with higher scores for a better state of health) according to standard methods; UK tariffs were used. The EQ-5D-5L questionnaire also included the EQ visual analog scale (EQ-VAS, range: 0–100, with higher scores for better quality of life). Functional impact and disability was evaluated by the self-reported Sheehan Disability Scale (SDS) (38). In the SDS, patients are asked to rate the extent to which (1) work, (2) social life or leasure activity, and (3) home life or family resposibilities are impaired on a 10-point visual analog scale, on which 0 is for normal function and 10 for severe funcional impairment; the total SDS score is calculated by summig up the 3 rates items, and thus ranges from 0 to 30, with higher scores for more severe impairment.

All pharmacological and non-pharmacological antidepressant therapies initiated at baseline and during the study period as for clinical practice were recorded.

Data on the healthcare resource utilization during the study period, including hospitalization, inpatient and outpatient visits, and intensive care unit (ICU) visits, were recorded.

### Data Processing and Analyses

Analyses were based mainly on data collected at baseline (available for all 124 patients) and at 6 months after baseline (available for 98 patients).

Antidepressant pharmacological treatment classes were categorized as: SSRI, SNRI, TCA, MAOI, and “other” (e.g., trazodone, bupropion, mirtazapine, vortioxetine). Non-antidepressant drugs were categorized as add-on or augmentation medications (e.g., quetiapine, brexpiprazole, aripiprazole, lithium). Combination therapy was defined as more than one antidepressant medication in the absence of add-on medication. Augmentation therapy was defined as antidepressant medication(s) plus at least one add-on medication.

We derived treatment lines being received by patients from baseline onward. Specifically, every treatment started at baseline (between 7 days before and 14 days after baseline) was defined as “first treatment line”. To be included in an appropriate “treatment line,” a treatment had to last at least 30 days: patients who started a treatment line at baseline but were lost to follow-up within 1 month and/or had no post-baseline visit with MADRS assessment were not taken into account in this analysis.

Remission and response status were defined according to the MADRS score. In particular, remission was defined as a MADRS score ≤10 and response (without remission) was defined as an improvement ≥50% from baseline in the MADRS score, with a MADRS >10.

The main study outcomes were the rates of patients with remission and response status at 6 and 12 months after baseline (only the assessments done during the visits which occurred, respectively, between days 150 and 216 and between days 330 and 402 after baseline were taken into account). Change in patient' condition according to the CGI-C score (*vs*. the baseline visit) at 6 month was also analyzed. Quality of life and functioning at 6 months were also described, overall and by clinical response.

### Statistical Analysis

Continuous variables were presented as mean values (±standard deviations) or median values (ranges), and categorical variables were reported as numbers and percentages. The Kaplan-Meier method was used to estimate the survival curve for time to treatment change.

## Results

In total, 124 Italian patients with TRD from 22 sites participated in the TRD European cohort and were included in the present analysis.

### Baseline Demographic Characteristics, Clinical Features and Medical History

At enrollment, the mean (SD) age was 53.2 (sd = 9.8) years and 82 patients (66.1%) were women (Table 1). Most patients had secondary (*n* = 50, 40.3%) or high school education (*n* = 44, 35.5%); 11 patients (8.9%) had University degrees. Seventy patients (56.5%) were married or had an official partner; 42 (33.9%) were employed, 52 (42.0%) unemployed and 17 (13.7%) were retired. The large majority of patients lived at home with cohabitants (*n* = 102, 82.3%); one patient lived at a psychiatric institution.

**Table 1:** Baseline socio-demographic characteristics and clinical features, psychiatric and medical history of patients with treatment-resistant depression (TRD).

	***N*** **= 124**
**Socio-demographic characteristics**	
Age (years), mean (sd) [range]	53.2 (9.8) [21–72]
Women, *n* (%)	82 (66.1)
**Education level**, ***n*** **(%)**	
Primary school	15 (12.1)
Secondary school	50 (40.3)
High school	44 (35.5)
University	11 (8.9)
Other/Unknown	4 (3.2)
**Marital status**	
Married/official partner	70 (56.5)
Not married/no official partner	31 (25.0)
Divorced	13 (10.5)
Widowed / Other	10 (8.0)
**Employed status**	
Employed	42 (33.9)
Unemployed	52 (42.0)
Retired	17 (13.7)
Other	13 (10.5)
**Living status**	
At home alone	21 (16.9)
At home with family, partner or friends	102 (82.3)
Psychiatric institution	1 (0.8)
**Clinical features**	
MADRS score, mean (sd) [range]	31.1 (6.1) [20–47]
MADRS score categories^a^, *n* (%)	
Moderate depression	88 (71.0)
Severe depression	36 (29.0)
**CGI-S score**, ***n*** **(%)**	
Mildly ill	5 (4.0)
Moderately ill	59 (47.6)
Markedly ill	47 (37.9)
Severely ill	12 (9.7)
Among the most extremely ill patients	1 (0.8)
**Psychiatric and medical history**	
Age (years) at MDD diagnosis, mean (sd)	37.3 (13.1)
Duration (years) of MDD, mean (sd)	16.0 (11.1)
First episode, *n* (%)	18 (14.5)
Previous depressive episodes, mean (sd)—median	5.2 (7.0) – 3.0
Duration (days) of current MDE, mean (sd)	97.5 (143.5)
**N. prior drug failures in current MDE**, ***n*** **(%)**	
2	75 (60.5)
3	35 (28.2)
≥4	14 (11.3)
**N. Ongoing comorbidities at baseline^b^**, ***n*** **(%)**	
0	69 (55.6)
1	23 (18.5)
2	18 (14.5)
≥3	14 (11.3)

a *Moderate depression, MADRS score 20–34; Severe depression, MADRS score >34*.

b *Twenty-nine patients reported endocrine/metabolic disorders, 28 cardiovascular diseases, 9 gastrointestinal diseases, 8 musculoskeletal diseases, 7 neurologic disorders, and 5 genito-urinary diseases; other comorbidities were reported by <4% of patients*.

*CGI-S, Clinical Global Impression of Severity; MADRS, Montgomery-Åsberg Depression Rating Scale; MDD, Major depressive disorder; MDE, major depressive episode; sd: standard deviation*.

The mean age at onset of MDD was 37.3 (sd = 13.1) years and the mean duration of the illness was 16.0 (sd = 11.1) years. The mean number of previous depressive episodes was 5.2, ranging from 0 to 43.

The current MDE had a mean duration of 97.5 (sd = 143.5) weeks, ranging from 13 to 1,304 weeks, and was a recurrent episode for over 85% of the patients; for treating it, 75 (60.5%), 35 (28.2%), and 14 (11.3%) patients had previously failed 2, 3 and ≥4 antidepressant drugs, respectively.

Fifty-five patients (44%) reported at least one ongoing comorbidity at baseline (32 patients 2 or more comorbidities), including both psychiatric and somatic ones; the most frequent ongoing conditions were endocrine and metabolic disorders (*n* = 29, 23.4%) and cardiovascular diseases (*n* = 28, 22.6%), followed by gastrointestinal (*n* = 9, 7.3%), musculoskeletal (*n* = 8, 6.5%), neurologic (*n* = 7, 5.6%), and genito-urinary diseases (*n* = 5, 4.0%). Other comorbidities were reported by <4% of patients.

The mean MADRS score at enrollment was 31.1 (sd = 6.1), with 88 patients (71.0%) being classified as having moderate depression (MADRS 20–34) and the remaining 36 (29.0%) as having severe depression (MADRS>34). Based on the CGI-S score (range: 1–7), 5 patients (4%) were categorized as mildly ill, 59 (47.6%) as moderately ill, 47 (37.9%) as markedly ill and 12 (9.7%) as severely ill; 1 patient had a CGI-S score of 7 (among the most extremely ill patients).

### Prior Failed Treatments and Successive Treatment Lines

**Figure 1:**
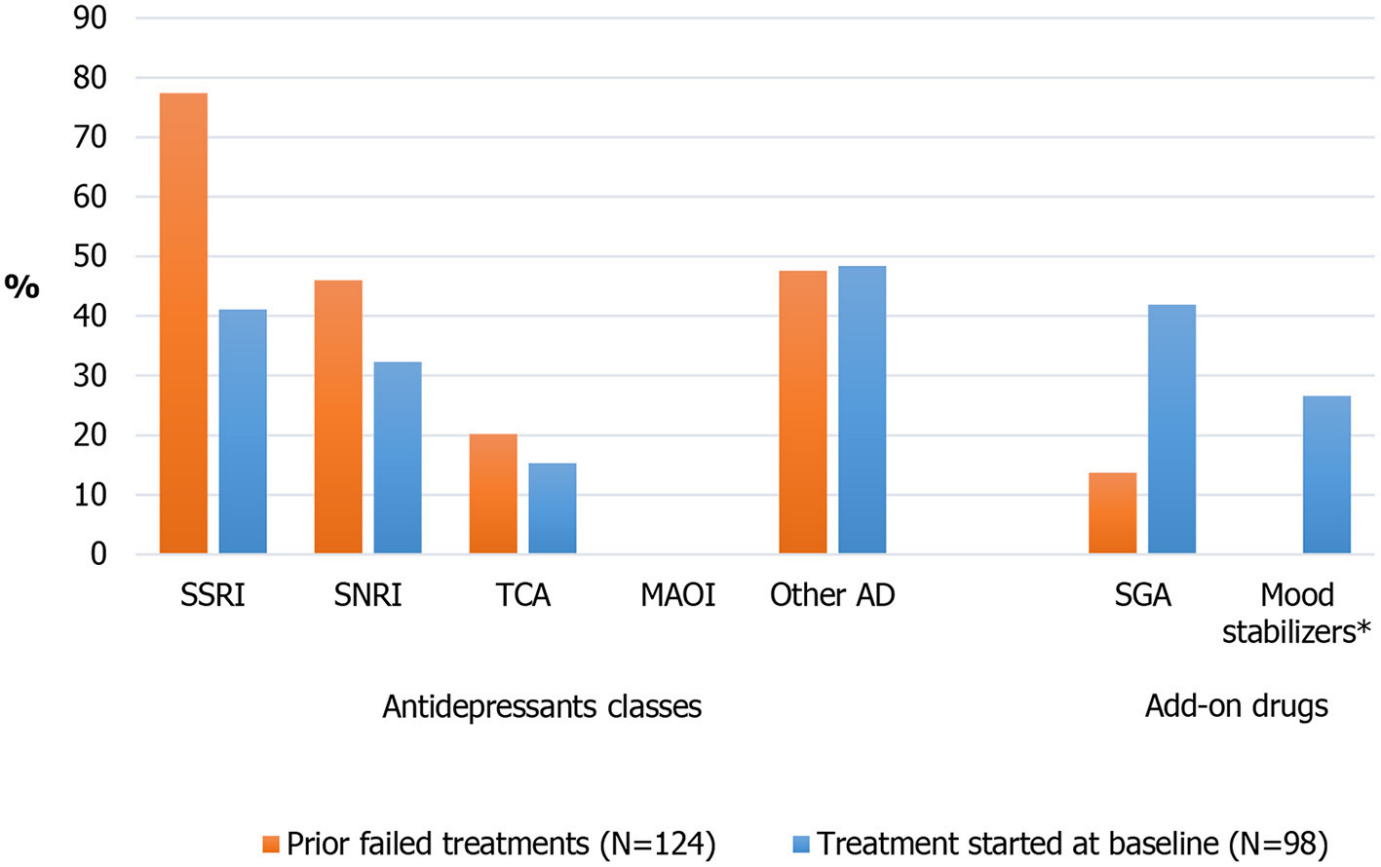
Prior failed antidepressive treatment and treatment started at baseline. *33 patients (26.6%) were treated with add-on mood stabilizers (16 patients [12.9%] with lithium) at baseline (not documented whether treatment onging at baseline or started at baseline). AD, antidepressants; MAOI, monoamine oxidase inhibitors; SGA, second-generation antipsychotics; SNRI, serotonin-norepinephrine reuptake inhibitors; SSRI, selective serotonin reuptake inhibitors; TCA, Tricyclic antidepressants.

The most frequent failed antidepressant classes were SSRI (*n* = 96, 77.4%) and SNRI (*n* = 57, 46.0%), followed by TCA (*n* = 25, 20.2%); prior add-on drugs (SGAs) were reported by 17 patients (13.7%) (Figure 1).

Information about drugs started at baseline was available for 98 patients (Figure 1): 42 patients (42.9%) started a SSRI (mainly sertraline, paroxetine, and escitalopram), 32 (32.7%) a SNRI (venlafaxine and duloxetine), 15 (15.3%) a TCA (mainly clomipramine), and 46 (46.9%) “other” antidepressant classes (including 23 patients [23.5%] receiving vortioxetine, 14 [14.3%] bupropion and 10 [10.2%] trazodone); 42 (42.9%) and 25 (25.5%) patients started, respectively, add-on SGAs (mostly quetiapine and aripiprazole) and add-on mood stabilizers (mostly lithium). No patient was treated with MAOI drugs. Regarding non-pharmacological approaches, no patient was treated with a neurostimulation approach. According to baseline information, 14 Italian patients (11.3%) had been treated with a psychosocial approach, including psychotherapy; in particualar, 7 patients (5.6%) received cognitive behavioral therapy.

In terms of treatment strategies, 50 patients (51.0%) started at baseline augmentation therapies, 18 (18.4%) combination therapies, and 24 (24.5%) monotherapies; 6 patients (6.1%) received a non-antidepressant drug (e.g., SGA) only.

According to Kaplan-Meier analysis, 73% of patients were still on the treatment received at baseline after 6 months (Supplementary Figure 1).

In the successive treatment line (*n* = 28), 19 patients (68%) received augmentation therapy; combination therapy, monotherapy and a non-antidepressant drug only were prescribed to 3 patients each (data not shown).

### Outcomes and Health Resource Utilization

**Figure 2:**
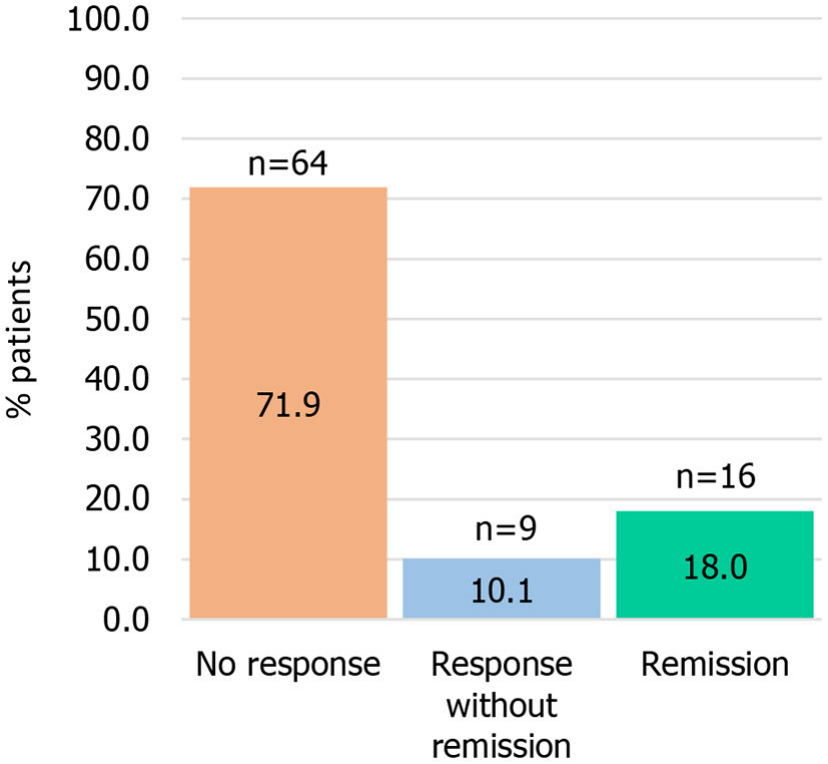
Treatment response^*^ among patients with treatment-resistant depression at 6 months. ^*^Remission: Montgomery-Åsberg Depression Rating Scale (MADRS) score ≤10; response (without remission): ≥50% improvement in the MADRS score, with MADRS>10.

At Month 6, 16 patients were reported as discontinued: 11 patients were lost to follow up; 2 died; 3 discontinued due to “other” reasons. Data were excluded from the 6 months outcome analysis for a further 18 patients who were still in the study but whose visits did not meet the defined cut-off dates for a Month 6 visit. We excluded one additional patient who had missing MADRS information at month 6. In total, 35 patients were not considered in the 6 month analysis (mean age: 52.2 years, % of women: 62.9%, mean MADRS: 29.9). All other 89 patients were included in this analysis, irrespective of treatment strategy. Among them, 64 (71.9%) showed no response, 9 (10.1%) response without remission and 16 (18.0%) remission (Figure 2). The MADRS score decreased, on average, by 31.6% (sd = 31.4), with a 6-month mean score of 21.5 (sd = 10.8): the mean score was 26.3 (sd = 8.6) in 64 non-responder patients, 13.8 (sd = 3.7) in 9 responder patients and 6.9 (SD 1.4) in 16 remitter patients.

At 6 months, 6 patients (6.7%) were classified as not at all ill, 10 (11.2%) as bordeline metally ill, 15 (16.9%) as mildly ill, 44 (49.4%) as moderately ill, 10 (11.2%) as markedly ill and 4 (4.5%) as severely ill based on the CGI-S score. The corresponding numbers were 0, 1, 12, 38, 9 and 4 in non-responder patients, 2, 1, 2, 3, 1, and 0 in responder patients, and 4, 8, 1, 3, 0, and 0 in remitter patients.

According to the CGI-C score, 30 patients improved much or very much (33.7%), 29 patients minimally improved (32.6%) and 30 (33.7%) did not benefit from treatment (no change or worse) at 6 months (Table 2). The means of EQ-5D-5L and EQ VAS were 0.44 (sd = 0.25) and 43.9 (sd = 19.0) at baseline and 0.6 (sd = 0.2) and 57.5 (sd = 22.3) at 6 months. The mean of the SDS scores decreased from 21.0 (sd = 5.7) at baseline to 15.0 (sd = 8.0) at 6 months. When patient-reported measures were analyzed according to clinical response, we found mean EQ-5D-5L scores of 0.6 in non-responder patients, 0.7 in responder patients and 0.9 in patients in remission; the corresponding mean values for EQ-VAS were, respectively, 50.1, 64.6 and 81.9 (Table 2). As for the disability, the mean SDS scores at 6 months was 18.6 in non-responder patients, 12.6 in responder patients, and 5.0 in patients with remission.

**Table 2:** Change in depression severity at 6 months compared to the baseline visit (CGI-C score), and quality of life and disability at baseline and at 6 months in patients with treatment-resistant depression (TRD), overall and by clinical response at 6 months.

	**Baseline**	**6 months**			
	**All patients *N =* 124**	**All patients *N =* 89**	**No response *N =* 64**	**Response without remission *N =* 9**	**Remission *N =* 16**
CGI-C score^*^, ***n*** **(%)**					
Very much improved		5 (5.6)	1 (1.6)	2 (22.2)	2 (17.6)
Much improved		25 (28.1)	9 (14.1)	3 (33.3)	13 (76.5)
Minimally improved		29 (32.6)	24 (37.5)	4 (44.4)	1 (5.9)
No change		18 (20.2)	18 (28.1)	0 (0)	0 (0)
Minimally worse		7 (7.9)	7 (10.9)	0 (0)	0 (0)
Much worse		5 (5.6)	5 (7.8)	0 (0)	0 (0)
EQ-5D-5L utility index, mean (sd) [*N*]	0.4 (0.3) [121]	0.6 (0.2) [85]	0.2 (0.2) [61]	0.7 (0.1) [8]	0.9 (0.1) [16]
EQ-VAS, mean (sd) [*N*]	43.9 (19.0) [123]	57.5 (22.3) [85]	50.1 (20.4) [61]	64.6 (15.0) [8]	81.9 (11.7) [16]
Total SDS, mean (sd) [*N*]	21.0 (5.7) [94]	15.0 (8.0) [61]	18.6 (5.9) [41]	12.6 (7.8) [7]	5.0 (4.0) [13]

*CGI-C, Clinical Global Impression of Change; EQ-5D-5L, EuroQol 5-dimension 5-level; EQ-VAS, EuroQol visual analog scale; N, n. of patients with available data; sd, standard deviation; SDS, Sheehan Disability Scale*.

* *compared with baseline visit*.

Outcomes data at the 12-months follow-up were available for 44 patients; 27 (61.4%) had no response, 7 (15.9%) achieved response without remission and 10 (22.7%) achieved remission (Supplementary Figure 2).

During the first 6 months of study, 11 patients (12.4%) were hospitalized, with a median of 11 (range: 1–34) days spent in hospital (Table 3). Sixty-three patients (70.8%) had at least one outpatient consultation.

**Table 3:** Healthcare resource utilization between baseline and month 6 in patients with treatment-resistant depression.

	***N*** **= 89**
Patients hospitalized, *n* (%)	11 (12.4)
N. of days in hospital^a^, median (range)	11 (1–34)
Patients visiting ICU, *n* (%)	4 (4.5)
Patients with day clinic visits, *n* (%)	6 (6.7)
Patients with night clinic visits, *n* (%)	1 (1.1)
Patients with outpatient consultation, *n* (%)	63 (70.8)

*ICU, intensive care unit*.

a *Among hospitalized patients*.

## Discussion

The present study provided original and interesting data on the characteristics, the naturalistic treatment pattern and the clinical outcome of a cohort of TRD patients with moderate to severe depression treated in Italian clinical practice. We also reported information on the health resource utilization of these patients.

A recent Italian study aimed at estimating the number of patients with TRD and at characterizing the pattern of drug use in the country (30). By applying TRD criteria by the EMA, the authors estimated over 101,000 Italian adults affected by TRD. They also reported frequent use of combination and augmentation therapy to treat the disease. That study was, however, a retrospective record-linkage study based on administrative databases and did not provided clinical outcome data (30). Collecting prospectively and *ad hoc* patient-level information on disease course, the current study represents, therefore, an important complementary source of data on TRD in Italy.

Our study showed the negative impact TRD has on patients in terms of employment status, quality of life and functioning, and documented the frequent use of SSRI and SNRI and of augmentation strategies in the everyday Italian clinical practice.

The moderate to severe MDE lasted on average almost 2 years, without any treatment response in more than 70% of the cases, suggesting a substantial disease burden over time. The poor response and remission rates observed at 6 and 12 months seem to reflect the difficult-to-treat nature of TRD: 71.9% of the patients were still non-responder to the treatment at month 6, with a mean MADRS score of 26.3, indicanting persistence of clinically relevant depressive symptoms. These results were similar to those observed in overall European cohort (34).

Given such negative outcomes, it is surprising that no patient in the Italian cohort was treated with neurostimulatory techniques and that the use of psychotherapy was very limited. In particular, psychosocial therapy was prescribed to 7% of our Italian patients but to 19.2% of patients in the overall Euoropean cohort (34). In a recent Danish study, psychotherapy was used in 18% of TRD patients (39). Our findings are aligned with the poor availability and spread of such techniques in Italian mental health departements; it is in any case worth to mention that information on the use of private psychotherapists is not available in the study.

In line with the overall European sample (33), at baseline, our patients had already suffered from MDD for over a decade. About 2 out of 3 of our patients were female, in line with the well-known sex ratio in the prevalence of depression (1). The mean duration of the MDE ongoing at baseline was almost 2 years and a considerable proportion of patients had experienced 3 or more previous drug failures during the ongoing MDE. A high proportion of patients, almost 50%, reported at least one ongoing comorbidity at baseline, either psychiatric or physical. The most frequent conditions were endocrine, metabolic disorders and cardiovascular diseases.

Long duration of current MDE together with the failure of several antidepressant treatments are main contributors to the high burden of disease of TRD condition, that is also associated with higher rates of psychiatric and physical comorbility, shorter life expectancy and higher mortality than non-TRD episodes (27, 28, 40, 41).

Confirming the high burden of TRD, our patients showed low quality of life and poor functioning, together with a substantial degree of disability. In particular, we observed a mean utility value derived from EQ-5D-5L of 0.44, similar to that reported in a recently published French TRD cohort (26). The much lower employment rate in our study (34%, which increased to 39% when calculated excluding retired patients) compared to that reported for the Italian population (63% among individuals aged 20–64 years in 2018 according to the Italian National Institute of Statistics) is not unexpected and confirms the personal and social burden of TRD. The negative impact of MDD in general, and TRD in particular, on work functioning, including employment status, has been widely reported in the literature (15, 42).

Describing the treatment approaches, we documented a widespread use of augmentation therapy, mostly with SGA, which increases as treatment changes. At enrolment, previously failed augmentation therapy was reported by ~14% of patients, while it was received by a considerable higher proportion of patient at baseline (51%) and at the successive treatment step (68%). An increase in the use of augmentation with successive treatment lines was reported also in the overall European cohort, but with lower prevalences (i.e., 36.7% at baseline and 47.2% at the successive treatment step) (34). Although a specific pharmacological treatment has not been established for TRD, augmentation is a mainstay in the pharmacotherapy of TRD and more severe/difficult to treat MDD (16), with multiple clinical trials and meta-analyses focused on the efficacy of SGA and lithium in MDD patients failing on an antidepressant (22, 23). Widespread use of a polypharmaceutical approach and augmentation in MDD was observed in a large, European, cross-sectional study, which also reported an association between augmentation/combination therapy and resistance to prior antidepressant therapy (43). In the only available Italian study on TRD 57% of patients were prescribed an add-on treatment starting from the third line of therapy (30).

An increasing prescription of SGA in MDD has been documented in the last years across various countries (44, 45), including European ones (46). Noteworthy, the SGA quetiapine got approval by the EMA as add-on medication to ongoing antidepressants in patients with MDD who have had sub-optimal response to treatment with other antidepressants [Quetiapine XR EPAR] (47). A recent meta-analysis showed a relatively limited efficacy of add-on treatment with SGA compared with placebo in TRD, with a pooled mean difference in the mean change from baseline in the MADRS score of 2.5 for SGA *vs*. placebo (48).

In our study, lithium augmentation was less frequently prescribed than SGA, as reported by others (43). Most of the available evidence is on the efficacy of lithium augmentation with TCAs, which are not widely used in contemporary clinical practice (49) (as also observed in the present study). On the contrary, the efficacy of lithium augmentation with SSRI and SNRI antidepressants is not well-documented (50). This observation, together with the diffuse negative perception of lithium side effects and safety concerns (51) and the need for monitoring of the serum lithium concentration and endocrine and renal function (50), may explain the lower use of lithium over SGA augmentation in clinical practice.

In terms of antidepressants, SSRI and SNRI represented the most frequently previously failed classes of antidepressants for the treatment of the index MDE as well as the treatments chosen most commonly at baseline in monotherapy or in combination; this indicates a still widespread use in clinical practice of these 2 antidepressant classes that are representative of the current Italian standard of care.

In general, we observed some improvements in depression severity, quality of life, functioning and disability at 6 months, with 32.6% of patients being classified as minimally improved, 28.1% as much improved and 5.6% as very much improved by the CGI-C score, and with scores of EQ-5D-5L and EQ-VAS increasing (respectively from 0.4 to 0.6 and from 43.9 to 57.5, on average) and scores of SDS decreasing (from 21.0 to 15.0, on average). However, response rates at 6 months were generally poor (remission: 19%, response: 10%); data at 12 months, limited to 44 patients with available information, did not demonstrate any meaningful improvement in outcomes with 6 additional months of treatment (remission: 15.9%, response: 22.7%). Notewhorty, disability evaluations with SDS and quality of life with EQ-5D-5L and EQ-VAS scores were consistent with MADRS outcomes, showing a substantial burden of disease in non-responder patients and suggesting a consistency between the physician's assessment of the disease and the patient's perception of their health and quality of life.

Although measures of function and quality of life are important secondary outcomes, remission, i.e., complete relief of depressive symptoms, is the most clinically relevant goal in MDD. Patients achieving remission have better functioning and prognosis and are much less likely to relapse than those achieving response with residual symptomatology (5). Low response and remission rates in TRD with standard care were reported by others (24, 25, 52). In particular, in a US, prospective, observational study of 124 TRD patients receiving treatment as usual, the 6-, 12- and 24-months remission (i.e., 30-item Inventory of Depressive Symptomatology-Self Report [IDS-SR-30] ≤14) rates were 2.5, 3.6, and 7.8%, respectively; the corresponding response (i.e, ≥50% IDS-SR-30 improvement) rates were, respectively, 8.4, 11.6, and 18.4% (24). Our results, consistently with those from other studies, underscore the unmet need for better treatment modalities for TRD, as the current options have a low chance to provide clinically desirable and long-term effects.

Because of the timeline of esketamine nasal spray approval (November 2019 by the EMA and March 2020 by the Italian Medicines Agency) and of its commercial availability in Italy (October 2020), our study did not capture data of this new treatment option for TRD patients, for which only data from RCT are available; it will be relevant to document its use and associated outcomes in more naturalistic settings.

This is the first real-world prospective study on TRD conducted in Italy; however, the sample size is relatively small compared to other studies. Among the other limitations, the diagnosis of MDD was made exclusively by clinical assessment according to standard criteria and no (semi)structured interview was administered. In addition, data collected in our study did not allow to associate outcomes to specific treatment strategies. Furthermore, outcomes data at 6 and 12 months of follow-up were available only for a proportion of patients; this was due to a number of reasons, including lost to follow up, patients drop out, study end (i.e., the study ended 6 months after the recruitment of the last patient), lack of MADRS and other clinical/patient reported outcome data, and the fact some patients did not have visits meeting criteria for a month 6 (i.e., occurred between day 150 and day 216 after baseline) or a month 12 visit (between day 330 and day 402). In particular, outcome data at 12 months were available for <40% of the originally recruited patients, and thus such results need to be interpreted with caution.

Our study has, however, major strengths, including the prospective design, the inclusion of patients encountered in routine clinical practice, the involvement of a number of sites across the various Italian Regions, and the *ad-hoc* prospective collection of clinical and patients reported outcomes through the observation period.

## Conclusions

Results from the present study clearly showed the negative impact of TRD in terms of chronicity of the symptomatology, impairment in quality of life and functioning, and documented the substantial burden experienced by real-world TRD patients in Italy as in other European Countries. We also documented the widespread use of SSRI and SNRI and of augmentation strategies and the unfrequent use of a psychosocial approach, including psychotherapy, in the everyday Italian clinical practice. The study shows that, despite the multiple treatment options, the disease remains a complex and challenging condition and suggests an unmet treatment need in TRD care.

## Data Availability Statement

The datasets presented in this article are not readily available because legal/commercial restriction. Requests to access the datasets should be directed to ddelmont@its.jnj.com.

## Ethics Statement

The studies involving human participants were reviewed and approved by Ethic Committees of the participating centers. The patients/participants provided their written informed consent to participate in this study.

## Author Contributions

DD, MA, GA, GP, and GR: substantial contributions to acquisition and analysis or interpretation of the data. GP, PC, SD, GR, AV, MA, GA, JM, and DD: drafting the article or revising it critically for important intellectual content. All authors contributed to the article and approved the submitted version.

## Conflict of Interest

GP received grant/research support from Angelini; speaker/consultant for Angelini, Janssen, Lundbeck, Neuraxpharm, Sanofi Aventis. GR received speaker/consultant fees for Angelini, Innova Pharma, Janssen, Lundbeck and Otsuka. AV received grant/research support and speaker/consultant fees for Angelini, Boheringer Ingelheim, Innovapharma, Janssen-Cilag, Lundbeck, Otsuka, Pfizer, Recordati, Roche, Rovi Pharma, Takeda. JM was employed by Janssen EMEA. MA was employed by Janssen-Cilag SpA. GA was employed by Janssen-Cilag SpA. DD was employed by Janssen-Cilag SpA. The remaining authors declare that the research was conducted in the absence of any commercial or financial relationships that could be construed as a potential conflict of interest. This article was based on the original study 54135419DEP4001 sponsored by Janssen EMEA. The funder had the following involvement with the study: study design, data collection and analysis, decision to publish, and preparation of the manuscript. Support for third-party writing assistance for this article, provided by Carlotta Galeone (Statinfo srl), was funded by Janssen-Cilag SpA in accordance with Good Publication Practice (GPP3) guidelines (http://www.ismpp.org/gpp3).

## Publisher's Note

All claims expressed in this article are solely those of the authors and do not necessarily represent those of their affiliated organizations, or those of the publisher, the editors and the reviewers. Any product that may be evaluated in this article, or claim that may be made by its manufacturer, is not guaranteed or endorsed by the publisher.
