# Supplementary material for: Clinical Features and Outcomes of 124 Italian Patients With Treatment Resistant Depression: A Real-World, Prospective Study
Source: Front Psychiatry. 2021 Nov 5;12:769693. doi: 10.3389/fpsyt.2021.769693 (PMC8603563; doi:10.3389/fpsyt.2021.769693)
Supplement: Supplementary file 1 [file Data_Sheet_1.PDF]

Supplementary material

Article: Clinical features and outcomes of 124 Italian patients with treatment resistant depression: a Real-World, prospective study

By Perugi et al.

Supplementary Figure S1. Kaplan-Meier curve for time to change therapy started at baseline*.

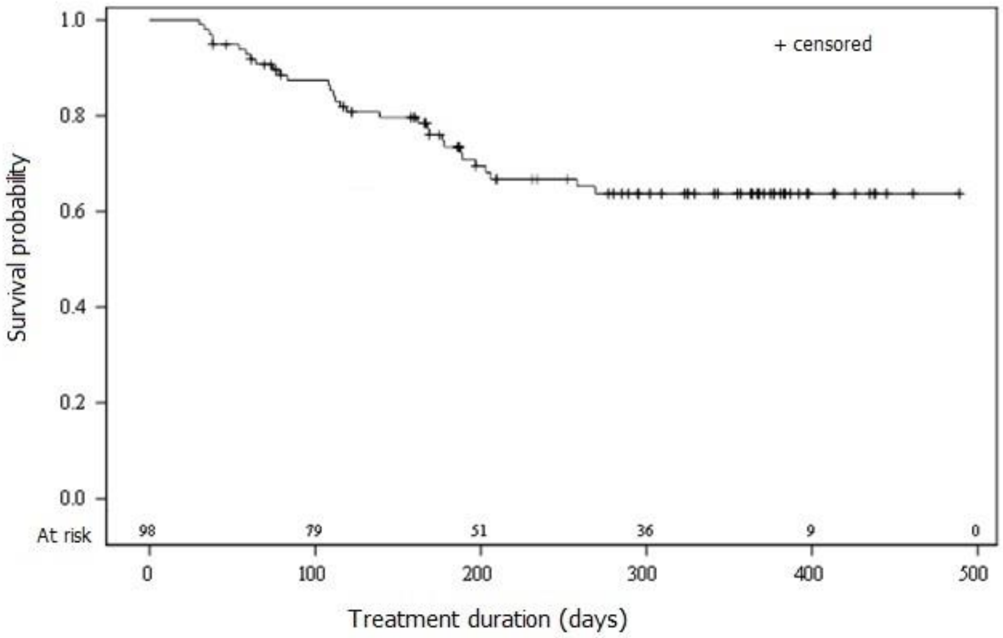

*Time to first treatment change from study entry (excluded treatment lines <30 days in duration and non-pharmacological treatments). Censoring was applied to treatments not stopped at the moment corresponding to the last study visit.

Supplementary Figure S2. Treatment response* among patients with treatment-resistant depression at 12 months.

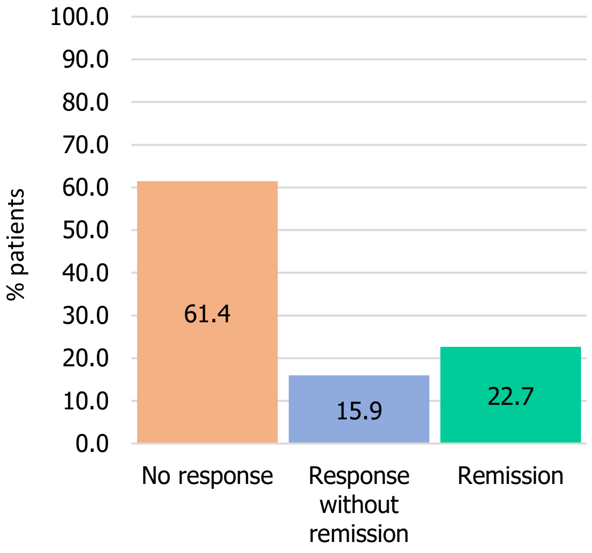

* Remission: Montgomery-Åsberg Depression Rating Scale (MADRS) score ≤10; response (without remission): ≥50% improvement in the MADRS score, with MADRS>10.
